# Supplementary material for: Prevalence and determinants of metabolic syndrome among long-shift healthcare professionals in primary hospitals of Central Gondar Zone, Northwest Ethiopia
Source: PLoS One. 2026 Jun 5;21(6):e0350807. doi: 10.1371/journal.pone.0350807 (PMC13240873; doi:10.1371/journal.pone.0350807)
Supplement: S2 Text — (DOCX) [file pone.0350807.s002.docx]

**English Version of the questionar**

Dear Participant, we thank you for agreeing to participate in the study titled “Prevalence and Determinants of Metabolic Syndrome Among Healthcare Professionals Working Long Shifts in Central Gondar Zone Primary Hospitals, Northwest Ethiopia: An Institutional-Based Cross-Sectional Study.” The aim of this study is to assess the prevalence of Metabolic Syndrome (MetS) and identify the associated factors among healthcare professionals working long shifts. Your valuable input will significantly contribute to a better understanding of the burden and determinants of MetS in this group, which is essential for developing targeted interventions to improve the health and well-being of healthcare professionals. Please note that participation is entirely voluntary. You have the right to refuse to answer any questions or withdraw from the study at any time without facing any consequences. Rest assured that all the information you provide will be kept strictly confidential and will only be used for research purposes.

If you have any questions or concerns about the study, please do not hesitate to contact the research team. Thank you once again for your valuable participation.

**Consent Statement**

By proceeding with this questionnaire, you confirm that you have been informed about the study and agree to participate.

**Are you willing to participate in the study?**

1. Yes 2. No

If yes, thanks!!! Conduct the interview. If the answer is no thanks!!!

Code--------- Name of data collector------------------ Signature ------- Date of interview-----

**Part I- Sociodemographic related questions**

| ***Q. N^o^*** | ***Questions*** | ***Answers Choices*** |
| --- | --- | --- |
| **A1** | How old are you? |  |
| **A2** | Sex |  |
| **A3** | What’s your average Monthly income in ETB? |  |
| **A4** | What is your current educational status? | 1. Diploma 2. First degree 3. Masters/MD/Higher |
| **A5** | How you describe your marital status? | 1. Single 2. Married 3. Widowed 4. Divorced 5. Other (Please specify) ……………… |
| **A6** | Profession | 1. Nurse 2. Medical doctor 3. Lab technologist 4. Pharmacist 5. Other (Please specify) ……………… |
| **A7** | Years of professional experience |  |

**Part II** – Anthropometric, and Clinical characteristics related question

| ***Q. No*** | **Questions** | **Answers** |
| --- | --- | --- |
| **B1** | Do you have a confirmed underlining medical condition? (“*A Medical condition means a health impairment or disease you are currently under treatment or living with*”) | 1. Yes 2. No (If your answer is “NO” please skip questions **B3**) |
| **B1.1** | Please specify the medical condition |  |
| **B3** | Do you have a family history of diabetes among your first-degree relatives (parents, siblings, or children)? | 1. Yes 2. No |
| **B4** | Do you have a family history of hypertension among your first-degree relatives (parents, siblings, or children)? | 1. Yes 2. No |
| **B5** | Do you have a family history of cardiovascular disease among your first-degree relatives (parents, siblings, or children)? | 1. Yes 2. No |
| **B6** | Do you frequently experience unexplained fatigue or low energy? | 1. Yes 2. No |
| **B7** | Do you experience frequent thirst or dry mouth? | 1. Yes 2. No |
| **B8** | Do you often feel excessively hungry, even after eating? | 1. Yes 2. No |
| **B9** | Have you noticed significant weight gain around your abdominal area? | 1. Yes 2. No |
| **B10** | Do you feel difficulty losing weight despite regular effort? | 1. Yes 2. No |
| **B11** | Do you experience frequent urination, especially at night? | 1. Yes 2. No |
| **B12** | Have you ever felt dizziness or lightheadedness after skipping a meal? | 1. Yes 2. No |
| ***For questions B13 – B17 measurements will be done by professionals*** | | |
| **B13** | **Body mass Index (BMI)________** | Weight cm |
|  |  | Height kg |
| **B14** | **Resting Blood pressure (BP)** | SBP______ mm/hg  DBP______ mm/hg |
| **B15** | **Waist to Hip ratio (WHR)_________** | Waist cm |
|  |  | Hip cm |
| **B16** | **Waist to Height ratio (WHtR)______** |  |
| **B17** | **MUAC** | -------------cm |

**Part III**– question related with Behavioral and Lifestyle characteristics

| ***No*** | **Questions** | **Answers** |
| --- | --- | --- |
| **C1** | On average, how many hours do you typically sleep at night??" |  |
| **C2** | How would you describe the quality of your sleep?  (*This refers to how often your sleep is disrupted, such as waking up during the night or having difficulty staying asleep*.) | 1. Uninterrupted sleep – meaning you would sleep through the night without waking up 2. Occasionally interrupted sleep – meaning you would wake up once or twice during the night but are able to fall back asleep 3. Frequently interrupted sleep – meaning you wake up multiple times during the night or have difficulty staying asleep for long periods |
| **C3** | Do you have a Coffee drinking habit?  (This refers to how often you consume coffee regardless of the amount) | 1. Rare/Never – You rarely drink coffee or do not consume it at all. 2. Weekly/Occasional – You drink coffee occasionally, such as a few times a week. 3. Daily/Regular – You drink coffee regularly, typically on a daily basis. |
| **C4** | Do you have a habit of practicing regular exercise?  (“*Physical exercise is doing any activity that increases heart rate once per day for 20–30 minutes as a continuous activity*”) | 1. Yes 2. No, I don’t   (If your answer is No, please skip **Q C5**) |
| **C4.1** | How often do you exercise? | 1. < 3 times/ week 2. > 3 times/ week |
| **C5** | Do you have a Smoking habit in the last 30 days?  (“*Smoking include the habit of consuming more than 2 cigarettes, cigars, or a pipe per week” and “Living with smoker include individuals who live with smoker in the same house for at least a month*”) | 1. Yes, I smoke or lives with smoker 2. No |
| **C6** | Do you drink alcohol?  (“*This refers to the frequency and amount of alcohol you typically consume; Drinking alcohol does not include drinking a few sips for religious purposes*”) | 1. Non-drinker – You do not consume alcohol at all. 2. Occasional drinker – You drink alcohol infrequently, such as on special occasions or a few times a month. 3. Moderate drinker – You drink alcohol regularly but in moderate amounts, typically adhering to health guidelines (e.g., up to 1–2 standard drinks per day). 4. Heavy drinker – You drink alcohol frequently and in larger quantities, often exceeding recommended health guidelines. |
| **C7** | Do you have Khat Chewing habit? | 1. Yes 2. No |
| **C8** | On the usual day, which of the following food groups have you consumed? (*Select all that apply*)" | 1. Grains, roots, and tubers (e.g., rice, bread, potatoes) 2. Pulses/legumes (e.g., lentils, beans, chickpeas) 3. Nuts and seeds 4. Dairy products (e.g., milk, yogurt, cheese) 5. Meat, poultry, and fish 6. Eggs 7. Dark green leafy vegetables 8. Other vegetables 9. Fruits 10. Fats and oils (e.g., butter, cooking oil) 11. Sweets (e.g., sugar, honey, candy) 12. Beverages (e.g., tea, coffee, juice) |
| **C9** | How many different food groups would you consume usually? | 1. 1–3 groups 2. 4–6 groups 3. 7 or more groups |
| **C10** | What is the primary type of food in your daily diet? | 1. Plant-based (e.g., grains, vegetables, legumes) 2. Mixed diet (e.g., plant-based and animal-based foods) 3. Animal-based (e.g., meat, fish, dairy) 4. Other (please specify): __________ |
| **C11** | How often do you consume fast food (e.g., burgers, pizza, fried chicken)? | 1. Rare/Never – You rarely or never eat fast food. 2. Occasional – You eat fast food a few times a month. 3. Frequent – You eat fast food several times a week or more. |
| **C12** | How often do you consume sugary foods (e.g., pastries, candies) or drinks (e.g., soda, sweetened beverages)? | 1. Rarely/ never 2. 1 - 3 times per week 3. 4 – 6 times per week 4. Daily |
| **C13** | What type of oil or fat is most commonly used for cooking in your household? (Select the option that best represents your primary choice.) | 1. Vegetable oil (e.g., sunflower, soybean, canola) 2. Animal fat (e.g., butter, lard) 3. Mixed oils or fats (e.g., a combination of vegetable oil and animal fat) 4. Other (please specify): __________ |

**Part IV-** Working condition and job-related questions

| ***No*** | **Questions** | **Answers** |
| --- | --- | --- |
| **D1** | What is your usual shift type at work? | 1. Day shift 2. Night shift 3. Rotational shift |
| **D2** | What is the typical duration of your shift? | 1. ≤ 8 hours 2. 8–12 hours 3. 12+ hours |
| **D3** | How many hours are you typically on duty per week? | 1. 40–48 hours 2. 49–54 hours 3. 55+ hours |
| **D4** | Over the past month, how often have you felt stressed at work?  (*Stress refers to feeling overwhelmed, unable to cope, or under pressure due to your work environment*.) | 1. Never 2. Rarely 3. Sometimes 4. Often 5. Always |
| **D5** | How satisfied are you with the following aspects of your job? (*Rate each on a scale of 1–5, where 1 = Very Dissatisfied and 5 = Very Satisfied*) | 1. Work environment: _____ 2. Work-life balance: _____ 3. Salary and benefits: _____ 4. Opportunities for professional growth: _____ 5. Relationships with colleagues: _____ |
| **D6** | *How would you rate your workload in the following areas? (Rate each on a scale of 1–10, where 1 = Very Low and 10 = Very High)* | 1. Mental demand: (How much mental effort does your work require?) _____ 2. Physical demand: (How physically demanding is your work?) _____ 3. Time pressure: (How hurried or rushed do you feel in completing tasks?) _____ 4. Effort: (How much effort is required to accomplish your job?) _____ 5. Frustration: (How frustrating or stressful is your work experience?) _____ |

***PART V –Laboratory reports***
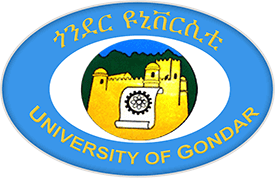


***University of Gondar comprehensive specialized hospital Laboratory unit***

**Client Information**

ID: _____________________

Age: _______________________

Sex: ________________________

Date of Sample Collection: ______

**Test Information**

Test Date: ___________________

Lab Technician: ___________________

Lab ID: __________________

Sample Type: _________________

Time of Sample Collection: ______

| **Biochemical profile I (Lipid profile tests)** | | ***Reference range (Beckman coulter)*** |
| --- | --- | --- |
| *Total cholesterol* | *mg/dl* | *<200 mg/dl* |
| *Triglyceride* | *mg/dl* | *<150 mg/dl* |
| *HDL-C* | *mg/dl* | *>50 mg/dl (Women) or >50 mg/dl (Men)* |
| *LDL-C* | *mg/dl* | *<130 mg/dl* |
| ***Biochemical profile II (Serum FBS)*** | |  |
| FBS | mg/dl | <126 *mg/dl* |
