# Supplementary material for: Prevalence and determinants of metabolic syndrome among long-shift healthcare professionals in primary hospitals of Central Gondar Zone, Northwest Ethiopia
Source: PLoS One. 2026 Jun 5;21(6):e0350807. doi: 10.1371/journal.pone.0350807 (PMC13240873; doi:10.1371/journal.pone.0350807)
Supplement: S3 Text — (DOCX) [file pone.0350807.s003.docx]

**Information sheet**

**Dear Sir/Madam,** my name is ____________, and I am conducting a research project titled **“***Prevalence and Determinants of Metabolic Syndrome Among Healthcare Professionals Working Long Shifts in Central Gondar Zone Primary Hospitals, Northwest Ethiopia: An Institutional-Based Cross-Sectional Study***.”** I kindly invite you to participate in this study.

***Please read the information below, and feel free to ask for clarification on any points before agreeing to participate.***

**Introduction**

This study is focused on assessing the *prevalence and determinants of metabolic syndrome* among healthcare professionals working long shifts in Central Gondar Zone Primary Hospitals, Northwest Ethiopia. Participation in this study is entirely voluntary. There will be no consequences if you choose not to participate or if you decide to stop completing the questionnaire at any point. It is your right to choose whether or not to participate in this study.

**What is expected from you as a participant?**

As a participant in this study, the following will occur:

- **Eligibility Screening:** We will review your clinical data to determine if you meet the eligibility criteria for the study.
- **Blood Sample:** You will be asked to provide a 5ml fasting blood sample, which will be used to analyze your serum lipid profile and fasting blood glucose levels.
- **Physical Measurements:** We will measure your blood pressure, mid-upper arm circumference (MUAC), weight, and height.
- **Questionnaire and Interview:** You will be asked to answer a series of questions in a questionnaire and participate in an interview. This information will contribute to the data for the study.

**Potential Benefits**

Your honest responses will help us better understand the metabolic abnormalities associated with long working shifts. You will also gain insights into your own metabolic health status, which includes key indicators like lipid levels and glucose levels. The study aims to identify major risk factors for metabolic syndrome that affect workplace quality and engagement. Ultimately, the findings could contribute to the prevention, early diagnosis, and treatment of metabolic syndrome, some aspects of which are modifiable.

**Compensation for Participation**

There will be no direct personal benefit or payment for your participation in this study, although the information gathered will be valuable in understanding metabolic syndrome and improving workplace health outcomes.

**Potential Risks**

The risks of participating in this study are minimal. You may experience mild discomfort or pain during the blood sample collection, but other than that, there are no significant risks associated with participation.

**Confidentiality**

All information collected will be kept confidential. We will use a coding system to ensure your data remains anonymous. Your blood sample, questionnaire responses, and laboratory results will all be associated with the same code, which will ensure your privacy. Only coded data will be analyzed, and no personal information will be shared.

**Contact Information**

If you have any questions or concerns during the study, please feel free to contact the principal investigator.

Thank you for considering participation in this important study. Please let me know if you have any questions or need further clarification.

**Consent Form**

**Code Number ……………**

I have read the information above, or it has been read to me. I have been given the opportunity to ask questions and my questions have been answered to my satisfaction. I voluntarily consent that I would participate in this study to collect my blood and be a participant in this study and understand that I have the right to withdraw from the study at any time.

Print code of participant, date and signature of participant

----------------/-----------/--------- (DD/MM/YY)

Print code of independent witness, date and signature of witness

----------------/-----------/--------- (DD/MM/YY)

Print name of researcher, date and signature of researcher

----------------/-----------/--------- (DD/MM/YY)

Data collector signature Date ________________
